# Non-ST-Segment Elevation Myocardial Infarction Shortly After Starting Steroid Replacement Therapy in a Patient With Adrenal Insufficiency

**DOI:** 10.7759/cureus.25061

**Published:** 2022-05-16

**Authors:** Mustafa Ahmed, Abdul Majeed Maliyakkal

**Affiliations:** 1 Medicine, Hamad Medical Corporation, Doha, QAT; 2 Clinical Medicine, QU Health, Qatar University, Doha, QAT; 3 Clinical Medicine, Weill Cornell Medicine-Qatar, Doha, QAT

**Keywords:** hydrocortisone, st-elevation myocardial infarction (stemi), glucocorticoid replacement, cardiovascular risk, adrenal insufficiency

## Abstract

Adrenal insufficiency is a rare disorder that results from etiological factors affecting either the hypothalamic-pituitary axis or the adrenal gland itself. Studies have associated an inherently increased risk of cardiovascular events with this condition. It is treated with exogenous steroid supplementation. However, in recent years, there have been an increasing number of reports regarding the potential of steroid therapy to precipitate acute cardiac events. However, this risk is generally assumed to be dose-dependent and could be absent in patients receiving low-dose glucocorticoid treatment. We present a case of a 71-year-old woman who was admitted to our institution with bilateral lower limb swelling. Blood investigation revealed hypoalbuminemia and hyponatremia. Upon further evaluation she was diagnosed to have adrenal insufficiency and was started on hydrocortisone replacement therapy; however, the patient developed non-ST-segment elevation myocardial infarction (NSTEMI) and acute pulmonary edema a few days after starting steroid replacement therapy. Here, we discuss the possible association between hydrocortisone use and the development of acute cardiac events.

## Introduction

Adrenal failure is an uncommon disorder that is estimated to occur in five in 10,000 individuals, with hypothalamic-pituitary disorder accounting for 60% and primary adrenal insufficiency (AI) accounting for the remaining 40% of cases [1]. Systemic glucocorticoid (GC) therapy, which is the main treatment modality, causes major side effects spanning multiple bodily systems, such as dermatological (acne, hirsutism, facial erythema, and striae), gastrointestinal (peptic ulcer disease, gastritis, steatohepatitis), bone and muscle (osteoporosis, avascular necrosis, and myopathy), endocrine (hypothalamic-pituitary-AI), metabolic (hyperglycemia, weight gain) and ophthalmic (cataract) effects. Other side effects include leukocytosis and an increased risk of infection. Its major cardiovascular side effects include fluid retention, hypertension, atherosclerosis, arrhythmia, and perturbations of serum lipoproteins. Moreover, GC therapy has been associated with increased cardiovascular events (angina or myocardial infarction requiring coronary revascularization, heart failure, transient ischemic attack, or stroke) [2,3]. However, this association seems to be dose-dependent [4]. Moreover, although little is known about cardiovascular disease in patients with adrenal failure, recent reports have linked adrenal failure and its treatment to acute cardiac events [5,6]. We report a newly diagnosed patient with AI who developed NSTEMI after starting steroid replacement therapy.

## Case presentation

 A 71-year-old woman with a history of type 2 diabetes, hypertension, osteoarthritis, morbid obesity, and depression. She also suffered from bronchial asthma and hypothyroidism (post-thyroidectomy) and was receiving steroid inhaler and thyroxin replacement therapy. She had parathyroidectomy with concomitant auto-transplantation of one gland in the left forearm many years ago, to manage primary hyperparathyroidism. She was admitted to our institution with a history of lower-limb edema for 10 days. Upon assessment, there were no signs of heart failure or chronic liver disease, but laboratory investigations revealed hyponatremia of 125 mmol/L and hypoalbuminemia of 25 g/L (Tables 1, 2).

**Table 1:** Laboratory results: hematology ANC: absolute neutrophil count; MCH: mean corpuscular hemoglobin; MCHC: mean corpuscular hemoglobin concentration; MCV: mean corpuscular volume; WBC: white blood cell count.

Lab test	During first admission	During second admission( 12 days after first admission)	Reference range
WBC	5.2	10.3	4–10 x 10^3^/µL
Hemoglobin	11.6	11.3	12–15 g/dL
MCV	88.3	98.4	83–101 fL
MCH	28.2	27.2	27–32 pg
MCHC	32	30.5	31.5–34.5 g/dL
Hematocrit	36.3	37.1	36%–46%
Platelets	250	410	150–400 x 10^3^/µL
ANC	1.2	7.3	2–7 x 10^3^/µL
Lymphocytes	2.79	2.03	1–3 x 10^3^/µL
Monocytes	0.81	0.95	0.2–1 x 10^3^/µL
Eosinophils	0.2	0.0	0–0.5 ´ 10^3^/µL
Basophils	0.05	0.02	0.02–0.10 x 10^3^/µL

 

**Table 2:** Laboratory results: chemistry and serology ACTH: adrenocorticotrophic hormone; NT-proBNP: N-terminal prohormone brain natriuretic peptide

Lab test	First admission	Second admission(12 days after first admission)	Reference range
Urea	1.8	2	3.5–7.2 mmol/L
Creatinine	67	56	50–98 µmol/L
Sodium	125	137	135–145 mmol/L
Potassium	4.1	4.3	3.6–5.1 mmol/L
Chloride	94	106	96–110 mmol/L
Bicarbonate	23	21	22–29 mmol/L
Adjusted calcium	2.28	2.3	2.10–2.55 mmol/L
Total protein	48	62	64–83 g/L
Albumin	26	35	35–50 g/L
Bilirubin	20.8	16.2	3.4–20.5 µmol/L
Aspartate aminotransferase	37	72	5–34 U/L
Alanine aminotransferase	25	35	0.0–55 U/L
Alkaline phosphatase	66	100	40–150 U/L
NT-proBNP	219.20	2705	7–137 pg/mL
Troponin T highly sensitive	14.58	492	0.0–14 ng/L
Random glucose	10.1	16.4	3.3–5.5 mmol/L
C-reactive protein	17.6	<5	0.0–5 g/dl
Hemoglobin A1C	7.3%	7.3%	4.8%–5.9%
Cholesterol	6.13	3.5	<5.2 mmol/L
Triglycerides	2.61	1.6	<1.7 mmol/L
High-density lipoprotein cholesterol	1.81		>1 mmol/L
Low-density lipoprotein cholesterol	3.15		<3.36 mmol/L
Treponema pallidum Ab	Non-reactive		
HIV Ag/Ab combo	Non-reactive		
Hepatitis B serology	Non-reactive		
Hepatitis C serology	Non-reactive		
Vitamin D	70		30–80 ng/mL
Thyroid-stimulating hormone	0.5		0.4–5.3 mIU/L
Free thyroxine	16.6		8.4–19.1 pmol/L
Parathyroid hormone	85		12–88 pg/mL
ACTH	51		7.2–63.3 pg/mL
Cortisol	23		185–642 nmol/L
Follicle-stimulating hormone	25.3		17–114 IU/L
Luteinizing hormone	9.6		11–59 IU/L
Prolactin	257.5		109–557 mIU/L
Insulin-like growth factor 1	97.7		54–161 µg/L

**Figure 1:**
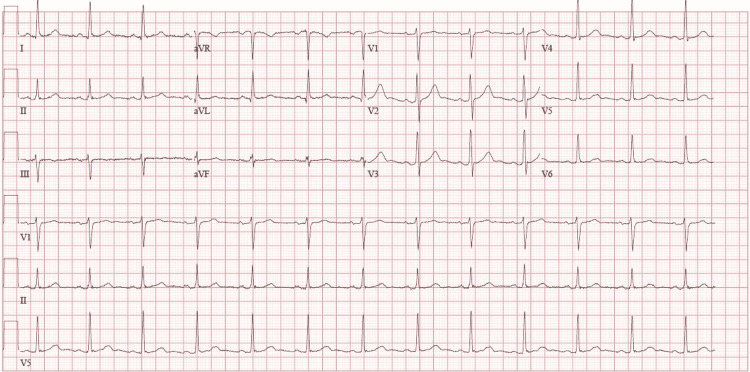
ECG performed during the patient's first admission, showing sinus rhythm

An abdominal ultrasound revealed the presence of fatty liver (with no evidence of cirrhosis) and increased renal parenchymal echogenicity; however, no signs of obstructive uropathy were observed. Electrocardiography (ECG) (Figure 1) showed a normal sinus rhythm with no ST-segment or T-wave changes. 

**Figure 2:**
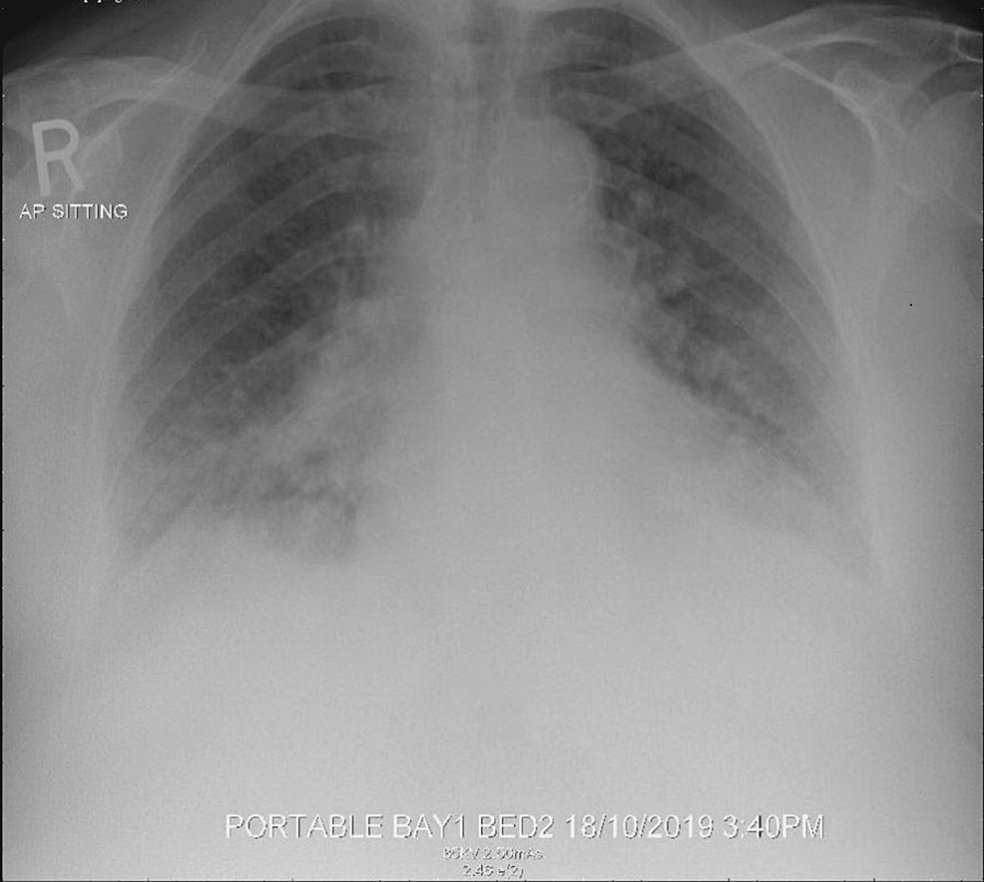
: Chest x-ray showing pulmonary edema on the patient’s second admission

**Figure 3:**
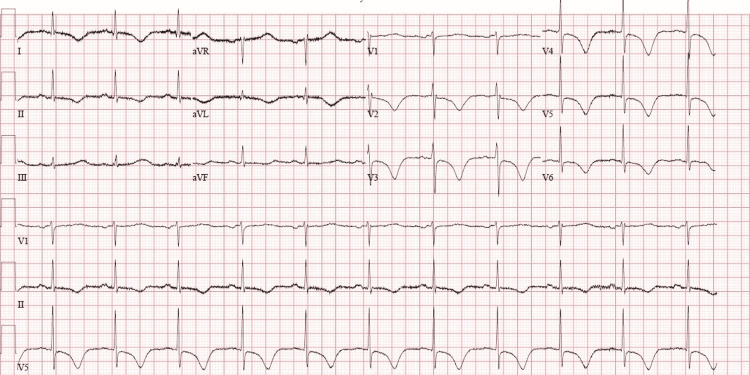
ECG showing deep T-wave inversion and ST-segment depression on the patient's second admission

Echocardiography revealed an ejection fraction of 68% with no regional wall motion abnormality. The N-terminal prohormone brain natriuretic peptide (NT-pro BNP) was 219.20 pg/mL (normal range, 7-137 pg/mL), and high-sensitive troponin T (hs-trop T) 14.58 ng/L (normal range, 0-14 ng/L). Urine analysis was normal, and the urine protein creatinine ratio was <22.6 mg/mmol. Her edema was suspected to be caused by hypoalbuminemia. The thyroid function test was normal. Her morning cortisol level was 23 nmol/L. A Synacthen test revealed a cortisol level of 82 nmol/L at baseline, 155 nmol/L at 30 min, and 183 nmol/L at 60 min. The adrenocorticotropic hormone (ACTH) level was 51.0 pg/mL (normal range, 7.2-63.3 pg/L). She was diagnosed with secondary AI and was started on hydrocortisone (HC) 15 mg q AM and 5 mg q PM. Prolactin (257.5 mIU/L) and insulin-like growth factor 1 (97.7 µg/L) were within the normal range. Although the level of the follicular stimulating hormone was within the normal range (25.3 IU/L) and that of the luteinizing hormone was low (9.6 IU/L), they were both considered inappropriately low as she was menopausal. The magnetic resonance imaging (MRI) head findings were in keeping with partial empty sella and chronic infarct in the right cerebellar hemisphere. A computerized tomography adrenal scan showed bilateral adrenal atrophy. The patient exhibited a remarkable improvement after commencing steroid therapy. The sodium level improved (140 mmol/L) and she had only mild lower-limb edema when she was discharged seven days later. Three days after discharge, the patient was readmitted with severe breathlessness and chest discomfort. She had bilateral basal crackles and suspected acute pulmonary edema/acute coronary syndrome (ACS). Chest x-ray showed pulmonary edema (Figure 2), and ECG revealed sinus rhythm inverted T-wave in anterolateral leads, which progressed to deep T-wave inversion in anterior leads within three hours (Figure 3).

 

Echocardiography showed significant changes compared with the previous echocardiography, that is, moderately reduced systolic left ventricular function (EF, 34%) and regional wall motion abnormalities. The level of hs-trop T was high (492.7 ng/L) and that of NT-pro BNP was significantly elevated (2,705.00 pg/mL). She was diagnosed as having NSTEMI and acute pulmonary edema. She was treated conservatively in the Medical Intensive Care Unit and received anti-ischemic and anti-failure treatment. With treatment, the patient improved remarkably, and her symptoms resolved. Repeat echocardiography showed an EF of 60% with no hypokinesia. Coronary angiography performed nine days after admission was normal. Finally, an MRI of the heart showed no myocardial infarction and no myocardial fibrosis, and the patient was discharged in good general condition.

## Discussion

AI is a rare condition, with secondary AI (hypothalamic-pituitary causes) accounting for most of the cases. The symptoms of primary and secondary AI are similar; however, hyperpigmentation is not present in secondary AI, and electrolytes are relatively normal (because of the normal functioning of the renin-angiotensin-aldosterone system). Hypoglycemia is commonly observed in secondary AI, especially in panhypopituitarism. The objectives of the treatment of AI are to control symptoms and prevention of adrenal crisis while on the lowest possible steroid replacement dose. According to a recent web-based survey, HC is the most commonly used replacement medication [7]. The average daily dose is 15-25 mg/day in both primary and secondary AI, usually divided into two or three doses [8,9]. HC has properties corresponding to endogenous cortisol bioavailability and receptor affinity [10]. Other steroids, such as prednisolone, may be utilized. Although a larger dose of HC is administered in the morning, to mimic the circadian rhythm, this often fails to mirror the exact physiological circadian rhythm, and four-dose regimens did not yield an improvement in quality of life [11,12]. The recently developed once-daily oral HC dual-release tablet holds the promise of improving the quality of life and metabolic measurements of patients, for example, waist circumference, HbA1c, and serum lipids [13]. Patients who are on replacement therapy appear to have high mortality. This has been proposed to be mainly caused by the cardiovascular risk (CVR) of GC replacement therapy [14-16]. HC at a dose >20 mg/day seems to increase CVR (9). A population-based study has shown an increased CVR and cerebrovascular risk in individuals taking GCs compared with matching controls without GC intake [17]. This could be attributed to the impact of GC therapy on CVR factors [11]. Patients receiving prednisolone tend to have higher LDL cholesterol compared with those receiving HC [17]. Endothelial dysfunction was proposed as a contributing factor to the increased mortality observed in patients taking high GC doses [18]. Increased inflammatory markers, for example, interleukin-6 (IL6), may contribute to the increased CVR detected in AI patients treated with conventional HC [19]. Moreover, AI has been reported in association with the acute coronary syndrome and acute myocardial infarction [20,21]. Functional hypoadrenalism may be associated with increased mortality and morbidity in critically ill patients.

Steroids have been reported to cause ST-segment elevation myocardial infarction (STEMI) [22]. Among the commonly used steroids, prednisolone at 40 mg was the only oral steroid reported to cause ACS, whereas other commonly used steroids were administered in the injectable form [23]. Our patient had multiple risk factors for cardiovascular disease, and similar risk factors were mentioned in most reported cases of steroid-induced STEMI [22]. The shortest reported time to onset was 7 min after the intravenous administration of 40 mg of methylprednisolone for anaphylaxis in a 20-year-old smoker [24]. Of note, prednisolone was reported to cause both NSTEMI and STEMI after a documented duration of use of one month [23]. Our patient had normal coronary angiography, despite the development of NSTEMI. The proposed mechanisms for myocardial infarction with “normal” coronary arteries include coronary vasospasm, coronary thrombosis in situ, embolization from a distal source with spontaneous lysis, cocaine abuse, viral myocarditis, aortic dissection, hypercoagulable states, autoimmune vasculitis, and carbon monoxide poisoning. Okumura et al. reported cases of steroid-induced coronary artery spasm, whereas Rogers et al. proposed that this may be attributed to decreased nitric oxide release, suppressed prostacyclin production, and increased synthesis of thromboxane [24,25]. Our patient, who was newly diagnosed with AI, was started on steroid replacement therapy and, within a few days (after 11 days), developed NSTEMI, presumably because of the freshly initiated HC therapy. Although HC has been reported to increase CVR at a dose >20 mg/day, our patient was receiving a dose of 20 mg/day [9]. We think that this case illustrates the importance of considering and educating the patient about the possibility of developing ACS after starting steroid replacement therapy.

## Conclusions

This case was unique in that the patient developed NSTEMI just a few days after the onset of HC replacement therapy for AI. To the best of our knowledge, no case of NSTEMI has been reported previously in association with HC replacement therapy for AI. Physicians should be aware of this possibility and educate their patients about its potential consequences (increased CVR), as well as the need for close monitoring in collaboration with their cardiologist.
